# Outpatient primary and tertiary healthcare utilisation among public rental housing residents in Singapore

**DOI:** 10.1186/s12913-019-4047-8

**Published:** 2019-04-15

**Authors:** Jun Jie Benjamin Seng, Vanessa Zi Kun Lim, Yu Heng Kwan, Julian Thumboo, Lian Leng LOW

**Affiliations:** 1 Duke-NUS Medical School, 8 College Road, Singapore, 169857 Singapore; 2 Program in Health Services and Systems Research, Duke-NUS Medical School, 8 College Road, Singapore, 169857 Singapore; 3 Health Services Research Centre, Singapore Health Services, Outram Road, Singapore, 169608 Singapore; 4 Department of Rheumatology and Immunology, Singapore General Hospital, Outram Road, Singapore, 169608 Singapore; 5 SingHealth Regional Health System, Singapore Health Services, 169608, Outram Road, Singapore, Singapore; 6 Department of Family Medicine and Continuing Care, Singapore General Hospital, Outram Road Singapore, Singapore, 169608 Singapore; 7 SingHealth Duke-NUS Family Medicine Academic Clinical Program, Outram Road, Singapore, 169608 Singapore

**Keywords:** Public rental housing, Ambulatory care, Primary healthcare utilisation, Public housing, Healthcare resources

## Abstract

**Background:**

Globally, public housing is utilized to provide affordable housing for low-income households. Studies have shown an association between public housing and negative health outcomes. There is paucity of data pertaining to outpatient primary and tertiary healthcare resources utilization among public rental housing residents in Singapore.

**Methods:**

A retrospective cohort study was performed, involving patients under the care of SingHealth Regional Health System (SHRS) in Year 2012. Healthcare utilization outcomes evaluated included number of outpatient primary and specialist care clinic visits, emergency department visits and hospitalization in Year 2011. Multivariate logistical analyses were used to examine the association between public rental housing and healthcare utilization.

**Results:**

Of 147,105 patients, 10,400 (7.1%) patients stayed in public rental housing. There were more elderly (54.8 ± 18.0 vs 49.8 ± 17.1, *p* < 0.001) and male patients [5279 (50.8%) vs 56,892 (41.6%), p < 0.001] residing in public rental housing. Co-morbidities such as hypertension and hyperlipidemia were more prevalent among public rental housing patients. (*p* < 0.05).

After adjustment for covariates, public rental housing was not associated with frequent outpatient primary care clinic or specialist outpatient clinic attendances (*p* > 0.05). However, it was associated with increased number of emergency department visits (OR: 2.41, 95% CI: 2.12–2.74) and frequent hospitalization (OR: 1.56, 95% CI: 1.33–1.83).

**Conclusion:**

Residing in public rental housing was not associated with increased utilization of outpatient healthcare resources despite patients’ higher disease burden and frequency of emergency department visits and hospitalizations. Further research is required to elucidate their health seeking behaviours.

**Electronic supplementary material:**

The online version of this article (10.1186/s12913-019-4047-8) contains supplementary material, which is available to authorized users.

## Background

Low socio-economic status (SES) has been shown to be associated with increased risk of illnesses and comorbidities [1]. In the United States, low income and education level have been found to predict increased risk for cardiovascular disease and mortality [2]. Low SES has also been demonstrated to influence patterns of utilization of healthcare services. While some studies found that lower SES groups encounter difficulties with regards to healthcare access [3, 4], they were shown to have higher healthcare utilization in countries where universal healthcare coverage is provided for [5, 6]. A study by Dani F. et al. found that people with lower SES tended to have more frequent emergency department visits and hospital admissions.

There exist a multitude of measures for SES which include factors such as education level, household income and occupation [7]. During routine clinical care, time may not permit for obtaining these details, which results in incomplete data [6]. Additionally, these data are not comprehensively captured at a population level. Housing type, which is available from patient’s home address and easily retrievable from electronic health records, may provide a more convenient measure of SES for physicians to screen patients.

Globally, public housing is utilized to provide affordable housing for low-income households [8, 9]. In Singapore, home ownership for majority of her 5.6 million citizens is achieved through public housing [10]. To help the lowest income (≤USD$1123 per month) households cope with living costs, one or two-room public flats are made available for rental from the government at heavily subsidized rates. These public rental housing flats are organized into blocks, and clusters of public rental housing blocks are located with public housing blocks of various types to promote neighbourhood social cohesion. However, studies have shown that residence in public housing is associated with negative health outcomes [11, 12]. Home ownership has been shown to have an inverse relationship with mortality [13]. In the HOPE VI panel study, public housing residents were found to have a two-fold higher likelihood of developing hypertension and hyperlipidaemia [14]. Another study showed that public housing is linked with obesity and poorer health statuses of mothers [11].

The significance of primary care and its contribution to a nation’s healthcare system is becoming increasingly recognized. Primary care is defined as essential healthcare made universally accessible to individuals in the community at an affordable cost that allows a continuing healthcare process [15]. As primary care serves as the first-line of care for most patients, the extent of utilization of primary care resources often reflects the population’s general health status and healthcare needs. Studies have also shown that people living in areas with more primary care physicians tend to have better health outcomes and that individuals’ utilization of primary care is associated with better health [16].

In Singapore, approximately 70–80% of its overall healthcare demands are addressed by the public sector [17]. Healthcare financing in Singapore primarily comprises of government subsidies and 3 flagship programmes which are namely Medisave, Medishield Life and Medifund [18, 19]. Every working Singaporean citizen contributes a proportion of their monthly salary to Medisave, a mandatory and government enforced medical savings account which pays for major healthcare expenditures such as hospitalization [19]. In contrast, Medishield Life is an automatically opt-in health insurance scheme which is used to subsidize high cost hospitalizations [19]. Lastly, Medifund is a means-tested social welfare program which is designed as a safety net to fund the healthcare costs of poorest citizens in the country, of which a significant proportion reside in public rental housing [19]. Therefore, out-of-pocket costs are expected to be minimal or nil for many residents living in public rental housing. A review by Chan et al. on health seeking behaviour of public rental housing residents found that they had lower participation in health screening, and preferred alternative medicine practitioners to western-trained doctors for primary care [20]. It is possible that many public rental housing residents may neglect health and primary healthcare due to conflicting life priorities, resulting in over-utilization of specialist and emergency care services at more advanced disease states.

Overall, the delivery of primary healthcare services locally is contributed by both private general practitioner (GP) clinics and public outpatient primary care clinics (polyclinics). While the majority (80%) of primary care provided by GPs in the private sector, polyclinics in each public regional health system play an important role in management and follow-up of 80% of patients with chronic diseases [21]. For patients with medical condition requiring specialist care, they are referred to outpatient specialist clinics located in tertiary centres. Although previous studies have examined the utilization of tertiary healthcare resources such as hospital services among public rental housing residents [6], there is no study which has examined their utilization of outpatient primary and tertiary healthcare resources in Singapore.

As such, this study aims to examine the utilization of outpatient primary and tertiary healthcare resources among public rental housing patients.

## Methods

A retrospective cohort study was conducted involving all adult patients who were under the medical care of SingHealth Regional Health System (SRHS) in Year 2012. Among the regional health systems in Singapore, SRHS is the largest cluster and is responsible for the provision of healthcare services to residents in South-Central Singapore We excluded patients who stayed in non-SRHS residential areas as they would fall under the purview of a different regional health system. Non-citizens were also excluded as the likelihood of them being under long-term medical care from SRHS is low. Approval from Singapore Health Services Centralized Institutional Review Board (CIRB 2016/2294) was obtained prior to the study’s initiation.

SRHS electronic medical records were utilized to extract patients’ socio-demographic and clinical details. These information included patient’s age, gender, ethnicity and the type of residential housing Major co-morbidities such as diabetes mellitus, hypertension and renal disease which are listed in the Charlson and Elixhauser comorbidity index were also collected [22]. The primary outcome in this study was the number of primary care clinic visits and specialist clinic visits that each patient had in Year 2011. Secondary outcomes that were examined included the number of emergency department visits and hospital admission for each patient in the past 1 year from date of inclusion. In this study, frequent primary care outpatient clinic attendance and outpatient specialist clinic visits were defined by ≥4 visits and ≥ 3 visits per year respectively [6]. Frequent emergency department visits and hospital admissions were defined as ≥4 visits and ≥ 3 admissions per year respectively [6, 23–26]. The cut-offs for frequent primary care outpatient clinic visits, outpatient specialist clinic visit and hospital admissions were determined by expert consensus across the major health regional systems in Singapore [23]. The threshold of ≥4 visits for emergency department visit as per a study performed by Locker et al. who found that there is > 99% of chance attenders who would presenting at the A&E on < 4 occasions per year as compared to a true frequent attender [24].

### Statistical analyses

Statistical analyses were performed using SPSS version 23 (SPSS Inc., Chicago, IL, USA). Differences in characteristics of patients who stayed and do not stay in public rental housing were assessed using Student’s t-test and Chi-square test, where appropriate. Univariate analyses were also performed to evaluate if there were differences in socio-demographic and clinical characteristics, as well as public rental housing residence status between patients with higher primary and tertiary healthcare utilization. Thereafter, variables with *p*-value < 0.05 were entered in the multivariate logistical regression model. A two-tailed p-value of < 0.05 was considered statistically significant.

## Results

**Fig. 1:**
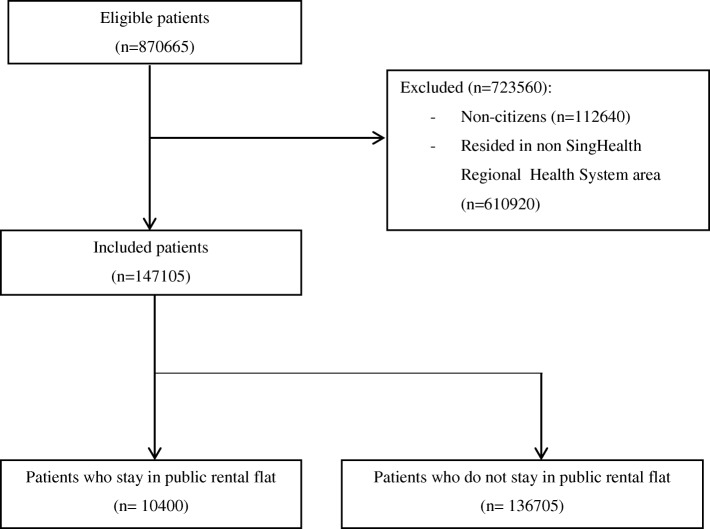
Flowchart for inclusion of patients

Figure 1 shows the flowchart for patient inclusion in the study. A total of 147,105 patients were included, of which 10,400 (7.1%) patients stayed in public rental housing.

Table 1 details the anthropomorphic and clinical characteristics of patients who stayed in public rental flats and not in public rental flat, as well as their utilization of healthcare resources among patients. The mean age of patients was 50.2 ± 17.2 years old and majority of patients were female (84,934, 57.7%). Compared to patients who did not stay in public rental housing, patients who stayed in public rental housing were older (54.8 ± 18.0 vs 49.8 ± 17.1, *p* < 0.001). In addition, there were more male [5279 (50.8%) vs 56,892 (41.6%), p < 0.001] but less Chinese [6367 (61.3%) vs 109,089 (79.8%), p < 0.001] patients staying in public rental flats relative to those patients not staying in public rental flats. The prevalence of most comorbidities such as diabetes, hypertension and hyperlipidemia were higher among patients staying in public rental flats (*p* < 0.05). However, there were no significant differences in the rates of hyperthyroidism, hypothyroidism, bipolar disease, anxiety as well as both non-metastatic and metastatic malignancy between the 2 groups of patients (*p* > 0.05). The attendance rates of polyclinics and hospital admissions were higher among patients who stayed in public rental housing (*p* ≤ 0.001). In contrast, patients who did not stay in public rental housing had higher number of outpatient specialist clinic visits (2.53 ± 5.66 vs 2.16 ± 5.47, *p* < 0.001).

**Table 1:** Patient characteristics and their association with residence in rental flats

	Stayed in public rental flat (*n* = 10,400)	Not stayed in public rental flat (*n* = 136,705)	All (*n* = 147,105)	*p* value
Patient Demographics in Year 2012				
Age, Mean (SD)	54.8 (18.0)	49.8 (17.1)	50.2 (17.2)	< 0.001
Gender				< 0.001
Female (%)	5121 (49.2)	79,813 (58.4)	84,934 (57.7)	
Male (%)	5279 (50.8)	56,892 (41.6)	62,171 (42.3)	
Ethnicity				< 0.001
Chinese (%)	6367 (61.3)	109,089 (79.8)	115,456 (78.5)	
Indian (%)	1185 (10.0)	10,078 (7.4)	11,263 (7.7)	
Malay (%)	2526 (24.3)	12,056 (8.8)	14,582 (9.9)	
Others (%)	322 (3.1)	5482 (4.0)	5804 (3.9)	
Deaths (%)	1063 (10.2)	5659 (4.1)	6722 (4.6)	< 0.001
Medical Comorbidities in Year 2012				
Diabetes without complications (%)	2023 (19.5)	18,785 (13.7)	20,808 (14.1)	< 0.001
Hypertension (%)	3983 (38.3)	39,074 (28.6)	43,057 (29.3)	< 0.001
Hyperlipidemia (%)	3503 (33.7)	38,934 (28.5)	42,437 (28.8)	< 0.001
Asthma (%)	708 (6.8)	4250 (3.1)	4958 (3.4)	< 0.001
Chronic Obstructive Pulmonary Disease (%)	700 (6.7)	2385 (1.7)	3085 (2.1)	< 0.001
Chronic Obstructive Pulmonary Disease with cor pulmonale (%)	603 (5.8)	1971 (1.4)	2574 (1.7)	< 0.001
Osteoarthritis (%)	1626 (15.6)	15,161 (11.1)	16,787 (11.4)	< 0.001
Hyperthyroidism (%)	69 (0.7)	1121 (0.8)	1190 (0.8)	0.086
Hypothyroidism (%)	132 (1.3)	1782 (1.3)	1914 (1.3)	0.766
Diabetes with complications (%)	279 (2.7)	1890 (1.3)	2169 (1.5)	< 0.001
Cerebrovascular accident (%)	689 (6.6)	4484 (3.3)	5173 (3.5)	< 0.001
Chronic Kidney Disease Stage 3–4 (%)	486 (4.7)	4128 (3.0)	4614 (3.1)	< 0.001
Chronic kidney disease stage V or End-stage renal failure (%)	268 (2.6)	1539 (1.8)	1807 (1.2)	< 0.001
Depression (%)	401 (3.9)	2409 (1.8)	2810 (1.9)	< 0.001
Schizophrenia (%)	151 (1.5)	410 (0.3)	561 (0.4)	< 0.001
Dementia (%)	63 (0.6)	450 (0.3)	513 (0.3)	< 0.001
Bipolar disease (%)	2 (0.02)	30 (0.02)	32 (0.02)	0.856
Anxiety (%)	102 (0.8)	1188 (0.9)	1290 (0.9)	0.239
Collagen vascular disease (%)	67 (1.0)	450 (0.3)	517 (0.4)	< 0.001
Parkinson disease (%)	50 (0.5)	431 (0.3)	481 (0.3)	0.004
Epilepsy (%)	127 (1.2)	588 (0.4)	715 (0.5)	< 0.001
Coronary heart disease (%)	1047 (10.1)	8462 (6.2)	9509 (6.5)	< 0.001
Atrial fibrillation (%)	150 (1.4)	1136 (0.8)	1286 (0.9)	< 0.001
Heart failure (%)	379 (3.6)	1187 (0.9)	2196 (1.5)	< 0.001
Peripheral vascular disease (%)	161 (1.6)	963 (0.7)	1124 (0.8)	< 0.001
Hip fracture (%)	53 (0.5)	226 (0.2)	279 (0.2)	< 0.001
Spine fracture (%)	71 (0.7)	381 (0.3)	452 (0.3)	< 0.001
Chronic liver disease (%)	137 (1.3)	937 (0.7)	1074 (0.7)	< 0.001
Pressure ulcer (%)	39 (0.4)	204 (0.2)	243 (0.2)	< 0.001
Non-metastatic malignancy (%)	365 (3.5)	4519 (3.3)	4884 (3.3)	0.268
Metastatic malignancy (%)	73 (0.7)	770 (0.6)	843 (0.6)	0.071
Past 1-year Healthcare Utilization in 2011				
Polyclinic attendances				
Number of visits, mean (SD)	3.12 (6.02)	2.40 (3.98)	2.45 (4.16)	0.001
Specialist outpatient clinic attendances				
Number of visits, mean (SD)	2.16 (5.47)	2.53 (5.66)	2.50 (5.65)	< 0.001
Emergency department attendances				
Number of visits, mean (SD)	0.43 (1.67)	0.13 (0.58)	0.15 (0.72)	< 0.001
Hospital admissions				
Number of visits, mean (SD)	0.26 (1.00)	0.11 (0.49)	0.12 (0.54)	< 0.001

Results for the univariate analyses for differences in characteristics of patients with more and less frequent primary care outpatient clinic, outpatient specialist clinic, emergency department visits and hospital admissions were reported in Additional files 1,2,3 and 4: Annexes A, B, C and D respectively in more detail (See Additional files 1,2,3 and 4: Annexes A, B, C, D).

Table 2 shows the results of multivariate analyses of public rental housing on healthcare utilization. After adjustment for socio-demographic and clinical covariates, public rental housing was associated with increased emergency department visits (OR: 2.41, 95% CI: 2.12–2.74) and frequent hospitalization (OR: 1.56, 95% CI: 1.33–1.83) but lower utilization of specialist outpatient clinics (OR: 0.83, 95% CI: 0.79–0.87). However, public rental housing was not associated with frequent outpatient primary care clinic attendances (OR: 1.048, 95% CI: 0.993–1.107).

**Table 2:** The impact of residing in public rental housing on hospital emergency utilization, outpatient primary care clinic, specialist outpatient clinic attendances and hospital admission in Year 2011

Outcomes	Residing in public rental housing OR (95% CI)	p-value
Frequent outpatient primary care clinic attendances (4 or more)	1.05 (0.99–1.11)	0.090
Frequent specialist outpatient clinic attendances	0.83 (0.79–0.87)	< 0.001
Frequent ED visits (3 or more)	2.41 (2.12–2.74)	< 0.001
Frequent hospital admission (3 or more)	1.56 (1.33–1.83)	< 0.001

Adjusted for Age, Gender, Ethnicity, Number of hospital admission, Specialist outpatient clinic visits, Emergency department visits (1 year before index admission), History of malignancy, Diabetes without complications, Hypertension, Hyperlipidemia, Chronic Kidney Disease Stage 3–4, Asthma, Chronic Obstructive Pulmonary Disease, Chronic Obstructive Pulmonary Disease with cor pulmonale, Osteoarthritis, Diabetes with complications, Cerebrovascular accident, Chronic kidney disease stage V or End-stage renal failure, Depression, Schizophrenia, Dementia, Collagen vascular disease, Parkinson disease, Epilepsy, Coronary heart disease, Atrial fibrillation, Heart failure, Peripheral vascular disease, Hip fracture, Spine fracture, Chronic liver disease, Pressure ulcer, Non-metastatic malignancy, Metastatic malignancy

## Discussion

In this study, residence in public rental housing was not associated with increased utilization of outpatient primary and tertiary healthcare resources despite their higher disease burden, increased number of emergency department and hospital admissions.

Studies examining primary care utilization by SES generally showed mixed results, where equitable distribution was observed across SES groups in some studies while other studies found increased usage of primary care services among low SES groups [27–29]. Increased primary healthcare utilization has been linked to better health outcomes [16] and it remains unclear if patients residing in public rental housing are utilizing primary healthcare resources optimally. This is especially of concern as they were found to have higher number of hospital admissions and emergency department visits.

Research has shown that there are many barriers to primary healthcare services utilization among lower SES groups. These barriers can largely be divided into categories which are namely population characteristics, patients’ cultural norms and values as well as healthcare system related services [30]. One of the cultural reasons that may compromise utilization of primary healthcare services is the perceived superiority of alternative medicine. A study showed that Western medicine were less preferred among lower income patients seeking primary healthcare, with only 11.1% preferring Western-trained physicians while 52.6 and 29.5% of patients prefer alternative medicine and self-reliance respectively [31]. The strong belief in self-reliance reflects a mindset that illnesses can get better without professional help and could impede early detection and treatment of diseases. Other patient and cultural related factors which may contribute to their low primary healthcare utilization includes misconception about health and financial costs [29, 32]. A study conducted among public rental housing residents found that they were more likely to seek medical attention only when symptoms such as pain manifest [33]. Another commonly cited health-system related reason suggested include lack of access to healthcare and long clinic waiting time [34]. It is noteworthy that a study by the English National Health Services found a 43-day difference in waiting time for non-coronary revascularization procedure between patients with different SES [35]. Locally, barriers that public rental housing residents commonly face for subsidized specialist care include the need to obtain referral letters from primary care physicians in public healthcare facilities and long waiting time, which can span up to three to six months [36].

It was interesting to note that public rental housing patients had lower utilization of specialist outpatient clinics. This was similar to findings from other studies that showed lower utilization of specialist visits in low SES groups compared to higher SES groups [5, 37]. A potential reason for this could be due to patients’ non-compliance with follow-up at SOCs. In contrast to primary care where services are typically provided within fixed-length appointment slots, specialists’ appointment lengths are highly variable and diagnosis-dependent, which may result in variable waiting times and inconvenience to patients [38]. Strong social support has also been shown to increase the probability of physician visits [39]. Although the level of social support among public rental housing residents was not assessed in this study, the lack of social support among public rental housing residents may potentially reduce their adherence to specialist outpatient clinic visits, especially among patients with ambulatory problems and should be explored in future studies.

The pattern of outpatient primary and tertiary outpatient healthcare utilization observed in this study could potentially be attributed to the heterogeneity in the health statuses and comorbidities of patients. With the paradigm shift towards greater efficiency for healthcare delivery, healthcare delivery targeted at groups of patients with similar pattern of healthcare utilization has been proposed [40]. Population segmentation via expert-driven and data-driven approaches has been suggested to identify healthcare needs of different patient groups. A local study that performed cluster analyses on a general population found that subjects could be segmented into 5 distinct clusters of patients with varying healthcare utilization and co-morbidities [41]. Future studies may wish to consider using population segmentation approaches to identify sub-groups of public rental housing patients which have overutilization or under-utilization of healthcare resources and poor health-related outcomes. This will aid the design of appropriate healthcare interventions to improve their health-related outcomes.

Pertaining to co-morbidities, public rental housing residents were found to have higher rates of co-morbidities such as depression and diabetes. Potential reasons for these findings could be due to circumstances surrounding their housing environment. Rental housing residents are often subjected to poorer housing conditions, where environmental hazards and poor hygiene may precipitate other illnesses [42]. Research has also shown that stressful life events are associated with heart disease, diabetes, major depression and other diseases [1]. Psychological stress comes about when an individual perceives tasks and demands to be exceeding his or her ability to cope [43]. Patients in low SES groups are at risk of higher psychological stress due to increased exposure to such stressors such as financial stress of supporting a family, poor social support and discrimination [7, 42, 44]. Metabolic diseases such as diabetes are commonly affected by diet and lifestyle choices. Individuals with lower SES have been shown to be less informed about implementing lifestyle changes in aspects of smoking, exercise and diet, as compared to their higher SES peers [1].

Overall, the higher disease burden among patients staying in public rental housing, coupled with their potential underutilization of outpatient primary and tertiary healthcare resources may explain their increased frequency of emergency department visits and hospitalizations. Further studies should be performed to understand the healthcare needs for patients residing in public rental housing as well as their health-seeking behaviours and attitudes to optimize their health-related outcomes.

This study is not without limitations. Firstly, data analysed in this study only included variables that were routinely collected from electronic databases within SHRS. Consequently, other measures of primary healthcare utilization such as healthcare related costs, health insurance claims and visits to private GP clinics could not be evaluated. Future studies may look into evaluating these measures as well as other socio-demographic characteristics (e.g. household income levels, marital status, family structure and support), attitudes and beliefs (e.g. self-reliant attitude, preference for alternative medicine) and barriers in knowledge (e.g. lack of access to information, misconceptions which may affect their healthcare utilization. Secondly, data pertaining to residents utilizing healthcare facilities in other regional health systems and non-users of the SRHS was unavailable, which may affect the representativeness of the reported population. However, it is expected that the proportion of residents utilizing facilities in other health systems to be small due to the geographical ease of access to the primary care facilities and specialist centres available in the SHRS. Lastly, a causal association between public rental housing and primary healthcare utilization could not be established due to the retrospective nature of the study.

## Conclusion

In this study, residing in public rental housing was not associated with increased utilization of outpatient primary and tertiary healthcare resources despite their higher disease burden and frequency of emergency department visits and hospitalizations. Further research is required to elucidate and understand health seeking behaviours among public-rental housing patients so as to optimize their appropriate utilization of outpatient primary and tertiary healthcare resources and improve their health-related outcomes.

## Additional files

Additional file 1: Annex A. Patient characteristics and their association with outpatient primary care clinic attendances. Annex A shows the univariate analyses results for differences in characteristics of frequent and non-frequent users of outpatient primary care clinics. (DOCX 17 kb)

Additional file 2: Annex B. Patient characteristics and their association with specialist outpatient clinic attendances. Annex B shows the univariate analyses results for differences in characteristics of frequent and non-frequent users of specialist outpatient clinics. (DOCX 17 kb)

Additional file 3: Annex C. Patient characteristics and their association with Emergency Department visits. Annex C shows the univariate analyses results for differences in characteristics of frequent and non-frequent users of emergency department. (DOCX 17 kb)

Additional file 4: Annex D. Patient characteristics and their association with frequent hospital admissions. Annex D shows the univariate analyses results for differences in characteristics of patients with frequent and non-frequent hospital admissions (DOCX 17 kb)

## Abbreviations

CI: Confidence interval
CIRB: Centralized Institutional Review Board
ED: Emergency department
GP: General practitioner
OR: Odds ratio
SD: Standard deviation
SES: Socioeconomic status
SHRS: SingHealth Regional Health System
SOC: Specialist outpatient clinic
USD: United States Dollar
