# Supplementary material for: Outpatient primary and tertiary healthcare utilisation among public rental housing residents in Singapore
Source: BMC Health Serv Res. 2019 Apr 15;19:227. doi: 10.1186/s12913-019-4047-8 (PMC6466644; doi:10.1186/s12913-019-4047-8)
Supplement: Supplementary file 1 — Annex A. Patient characteristics and their association with outpatient primary care clinic attendances. Annex A shows the univariate analyses results for differences in characteristics of frequent and non-frequent users of outpatient primary care clinics. (DOCX 17 kb) [file 12913_2019_4047_MOESM1_ESM.docx]

Additional file 1

**Annex A:** Patient characteristics and their association with outpatient primary care clinic attendances

|  | **Frequent polyclinic user**  **(n=42515)** | **Non-frequent polyclinic user**  **(n=104590)** | **All**  **(n= 147105)** | **p value** |
| --- | --- | --- | --- | --- |
| **Patient Demographics** | | | | |
| Age, Mean (SD) | 58.71 (15.88) | 45.26 (16.21) | 50.2 (17.2) | <0.001 |
| Gender |  |  |  | <0.001 |
| Female (%) | 23656 (55.6) | 61278 (58.6) | 84934 (57.7) |  |
| Male (%) | 18859 (44.4) | 43312 (41.4) | 62171 (42.3) |  |
| Ethnicity |  |  |  | <0.001 |
| Chinese (%) | 34618 (81.4) | 80838 (77.3) | 115456 (78.5) |  |
| Indian (%) | 2979 (7.0) | 8284 (7.9) | 11263 (7.7) |  |
| Malay (%) | 3841 (9.0) | 10741 (10.3) | 14582 (9.9) |  |
| Others (%) | 1077 (2.5) | 4727 (4.5) | 5804 (3.9) |  |
| Resided in public rental housing | 3524 (8.3) | 6876 (6.6) | 10400 (7.1) | <0.001 |
| **Medical Comorbidities** | | | | |
| Diabetes without complications (%) | 15357 (36.1) | 5451 (5.2) | 20808 (14.1) | <0.001 |
| Hypertension (%) | 29231 (68.8) | 13826 (13.2) | 43057 (29.3) | <0.001 |
| Hyperlipidemia (%) | 29432 (69.2) | 13005 (12.4) | 42437 (28.8) | <0.001 |
| Asthma (%) | 2445 (5.8) | 2514 (2.4) | 4958 (3.4) | <0.001 |
| Chronic Obstructive Pulmonary Disease (%) | 1377 (3.2) | 1708 (1.6) | 3085 (2.1) | <0.001 |
| Chronic Obstructive Pulmonary Disease with cor pulmonale (%) | 1031 (2.4) | 1543 (1.5) | 2574 (1.7) | <0.001 |
| Osteoarthritis (%) | 9127 (21.5) | 7660 (7.3) | 16787 (11.4) | <0.001 |
| Hyperthyroidism (%) | 732 (1.7) | 458 (0.4) | 1190 (0.8) | <0.001 |
| Hypothyroidism (%) | 1479 (3.5) | 435 (0.4) | 1914 (1.3) | <0.001 |
| Diabetes with complications (%) | 1720 (4.0) | 449 (0.4) | 2169 (1.5) | <0.001 |
| Cerebrovascular accident (%) | 2983 (7.0) | 2190 (2.1) | 5173 (3.5) | <0.001 |
| Chronic Kidney Disease Stage 3-4 (%) | 3050 (7.2) | 1564 (1.5) | 4614 (3.1) | <0.001 |
| Chronic kidney disease stage V or End-stage renal failure (%) | 694 (1.6) | 1113 (1.1) | 1807 (1.2) | <0.001 |
| Depression (%) | 1203 (2.8) | 1607 (1.5) | 2810 (1.9) | <0.001 |
| Schizophrenia (%) | 280 (0.7) | 281 (0.3) | 561 (0.4) | <0.001 |
| Dementia (%) | 247 (0.6) | 266 (0.3) | 513 (0.3) | <0.001 |
| Bipolar disease (%) | 12 (0.03) | 20 (0.02) | 32 (0.02) | 0.329 |
| Anxiety (%) | 585 (1.4) | 705 (0.7) | 1290 (0.9) | <0.001 |
| Collagen vascular disease (%) | 152 (0.4) | 365 (0.3) | 517 (0.4) | 0.811 |
| Parkinson disease (%) | 252 (0.6) | 229 (0.2) | 481 (0.3) | <0.001 |
| Epilepsy (%) | 286 (0.7) | 429 (0.4) | 715 (0.5) | <0.001 |
| Coronary heart disease (%) | 5779 (13.6) | 3730 (3.6) | 9509 (6.5) | <0.001 |
| Atrial fibrillation (%) | 544 (1.3) | 742 (0.7) | 1286 (0.9) | <0.001 |
| Heart failure (%) | 1003 (2.4) | 1193 (1.1) | 2196 (1.5) | <0.001 |
| Peripheral vascular disease (%) | 570 (1.3) | 554 (0.5) | 1124 (0.8) | <0.001 |
| Hip fracture (%) | 96 (0.2) | 183 (0.2) | 279 (0.2) | 0.046 |
| Spine fracture (%) | 198 (0.5) | 254 (0.2) | 452 (0.3) | <0.001 |
| Chronic liver disease (%) | 532 (1.3) | 542 (0.5) | 1074 (0.7) | <0.001 |
| Pressure ulcer (%) | 71 (0.2) | 172 (0.2) | 243 (0.2) | 0.947 |
| Non-metastatic malignancy (%) | 1644 (3.9) | 3240 (3.1) | 4884 (3.3) | <0.001 |
| Metastatic malignancy (%) | 224 (0.5) | 619 (0.6) | 843 (0.6) | 0.138 |
