# Supplementary material for: Outpatient primary and tertiary healthcare utilisation among public rental housing residents in Singapore
Source: BMC Health Serv Res. 2019 Apr 15;19:227. doi: 10.1186/s12913-019-4047-8 (PMC6466644; doi:10.1186/s12913-019-4047-8)
Supplement: Supplementary file 2 — Annex B. Patient characteristics and their association with specialist outpatient clinic attendances. Annex B shows the univariate analyses results for differences in characteristics of frequent and non-frequent users of specialist outpatient clinics. (DOCX 17 kb) [file 12913_2019_4047_MOESM2_ESM.docx]

Additional file 2

**Annex B:** Patient characteristics and their association with specialist outpatient clinic attendances

|  | **Frequent SOC user**  **(n=38608)** | **Non-frequent SOC user**  **(n=108497)** | **All**  **(n= 147105)** | **p value** |
| --- | --- | --- | --- | --- |
| **Patient Demographics** | | | | |
| Age, Mean (SD) | 53.21 (17.28) | 47.70 (16.98) | 50.2 (17.2) | <0.001 |
| Gender |  |  |  | <0.001 |
| Female (%) | 25181 (65.2) | 59753 (55.1) | 84934 (57.7) |  |
| Male (%) | 13427 (34.8) | 48744 (44.9) | 62171 (42.3) |  |
| Ethnicity |  |  |  | <0.001 |
| Chinese (%) | 31705 (82.1) | 83751 (77.2) | 115456 (78.5) |  |
| Indian (%) | 2974 (7.7) | 8289 (7.6) | 11263 (7.7) |  |
| Malay (%) | 2563 (6.6) | 12019 (11.1) | 14582 (9.9) |  |
| Others (%) | 1366 (3.5) | 4438 (4.1) | 5804 (3.9) |  |
| Resided in public rental housing | 2322 (6.0) | 8078 (7.4) | 10400 (7.1) | <0.001 |
| **Medical Comorbidities** | | | | |
| Diabetes without complications (%) | 6879 (17.8) | 13929 (12.8) | 20808 (14.1) | <0.001 |
| Hypertension (%) | 14336 (37.1) | 28721 (26.5) | 43057 (29.3) | <0.001 |
| Hyperlipidemia (%) | 13741 (35.6) | 28696 (26.4) | 42437 (28.8) | <0.001 |
| Asthma (%) | 1479 (3.8) | 3479 (3.2) | 4958 (3.4) | <0.001 |
| Chronic Obstructive Pulmonary Disease (%) | 1439 (3.7) | 1646 (1.5) | 3085 (2.1) | <0.001 |
| Chronic Obstructive Pulmonary Disease with cor pulmonale (%) | 1319 (3.4) | 1255 (1.2) | 2574 (1.7) | <0.001 |
| Osteoarthritis (%) | 6170 (16.0) | 10617 (9.8) | 16787 (11.4) | <0.001 |
| Hyperthyroidism (%) | 395 (1.0) | 795 (0.7) | 1190 (0.8) | <0.001 |
| Hypothyroidism (%) | 692 (1.8) | 1222 (1.1) | 1914 (1.3) | <0.001 |
| Diabetes with complications (%) | 945 (2.4) | 1224 (1.1) | 2169 (1.5) | <0.001 |
| Cerebrovascular accident (%) | 2157 (5.6) | 3016 (2.8) | 5173 (3.5) | <0.001 |
| Chronic Kidney Disease Stage 3-4 (%) | 2429 (6.3) | 2185 (2.0) | 4614 (3.1) | <0.001 |
| Chronic kidney disease stage V or End-stage renal failure (%) | 1408 (3.6) | 399 (0.4) | 1807 (1.2) | <0.001 |
| Depression (%) | 1070 (2.8) | 1740 (1.6) | 2810 (1.9) | <0.001 |
| Schizophrenia (%) | 190 (0.5) | 371 (0.3) | 561 (0.4) | <0.001 |
| Dementia (%) | 262 (0.7) | 251 (0.2) | 513 (0.3) | <0.001 |
| Bipolar disease (%) | 20 (0.1) | 12 (0.01) | 32 (0.02) | <0.001 |
| Anxiety (%) | 500 (1.3) | 790 (0.7) | 1290 (0.9) | <0.001 |
| Collagen vascular disease (%) | 386 (1.0) | 131 (0.1) | 517 (0.4) | <0.001 |
| Parkinson disease (%) | 274 (0.7) | 207 (0.2) | 481 (0.3) | <0.001 |
| Epilepsy (%) | 264 (0.7) | 451 (0.4) | 715 (0.5) | <0.001 |
| Coronary heart disease (%) | 4491 (11.6) | 5018 (4.6) | 9509 (6.5) | <0.001 |
| Atrial fibrillation (%) | 918 (2.4) | 368 (0.3) | 1286 (0.9) | <0.001 |
| Heart failure (%) | 1427 (3.7) | 769 (0.7) | 2196 (1.5) | <0.001 |
| Peripheral vascular disease (%) | 755 (2.0) | 369 (0.3) | 1124 (0.8) | <0.001 |
| Hip fracture (%) | 181 (0.5) | 98 (0.1) | 279 (0.2) | <0.001 |
| Spine fracture (%) | 258 (0.7) | 194 (0.2) | 452 (0.3) | <0.001 |
| Chronic liver disease (%) | 623 (1.6) | 451 (0.4) | 1074 (0.7) | <0.001 |
| Pressure ulcer (%) | 172 (0.4) | 71 (0.1) | 243 (0.2) | <0.001 |
| Non-metastatic malignancy (%) | 3815 (9.9) | 1069 (1.0) | 4884 (3.3) | <0.001 |
| Metastatic malignancy (%) | 803 (2.1) | 40 (0.04) | 843 (0.6) | <0.001 |
