# Supplementary material for: Outpatient primary and tertiary healthcare utilisation among public rental housing residents in Singapore
Source: BMC Health Serv Res. 2019 Apr 15;19:227. doi: 10.1186/s12913-019-4047-8 (PMC6466644; doi:10.1186/s12913-019-4047-8)
Supplement: Supplementary file 3 — Annex C. Patient characteristics and their association with Emergency Department visits. Annex C shows the univariate analyses results for differences in characteristics of frequent and non-frequent users of emergency department. (DOCX 17 kb) [file 12913_2019_4047_MOESM3_ESM.docx]

Additional file 3

**Annex C:** Patient characteristics and their association with Emergency Department visits

|  | **Frequent ED admitters**  **(n=1484)** | **Non-frequent ED admitters**  **(n=145621)** | **All**  **(n= 147105)** | **p value** |
| --- | --- | --- | --- | --- |
| **Patient Demographics** | | | | |
| Age, Mean (SD) | 56.37 (20.81) | 49.07 (17.18) | 50.2 (17.2) | <0.001 |
| Gender |  |  |  | <0.001 |
| Female (%) | 683 (46.0) | 84251 (57.9) | 84934 (57.7) |  |
| Male (%) | 801 (54.0) | 61370 (42.1) | 62171 (42.3) |  |
| Ethnicity |  |  |  | <0.001 |
| Chinese (%) | 1022 (68.9) | 114434 (78.6) | 115456 (78.5) |  |
| Indian (%) | 241 (16.2) | 11022 (7.6) | 11263 (7.7) |  |
| Malay (%) | 163 (11.0) | 14419 (9.9) | 14582 (9.9) |  |
| Others (%) | 58 (3.9) | 5746 (3.9) | 5804 (3.9) |  |
| Resided in public rental housing | 392 (26.4) | 10008 (6.9) | 10400 (7.1) | <0.001 |
| **Medical Comorbidities** | | | | |
| Diabetes without complications (%) | 496 (33.4) | 20312 (13.9) | 20808 (14.1) | <0.001 |
| Hypertension (%) | 840 (56.6) | 42217 (29.0) | 43057 (29.3) | <0.001 |
| Hyperlipidemia (%) | 738 (49.7) | 41699 (28.6) | 42437 (28.8) | <0.001 |
| Asthma (%) | 203 (13.7) | 4755 (3.3) | 4958 (3.4) | <0.001 |
| Chronic Obstructive Pulmonary Disease (%) | 315 (21.2) | 2770 (1.9) | 3085 (2.1) | <0.001 |
| Chronic Obstructive Pulmonary Disease with cor pulmonale (%) | 307 (20.7) | 2267 (1.6) | 2574 (1.7) | <0.001 |
| Osteoarthritis (%) | 398 (26.8) | 16389 (11.3) | 16787 (11.4) | <0.001 |
| Hyperthyroidism (%) | 4 (0.3) | 1186 (0.8) | 1190 (0.8) | 0.013 |
| Hypothyroidism (%) | 30 (2.0) | 1884 (1.3) | 1914 (1.3) | 0.016 |
| Diabetes with complications (%) | 110 (7.4) | 2059 (1.4) | 2169 (1.5) | <0.001 |
| Cerebrovascular accident (%) | 274 (18.5) | 4899 (3.4) | 5173 (3.5) | <0.001 |
| Chronic Kidney Disease Stage 3-4 (%) | 310 (20.9) | 4304 (3.0) | 4614 (3.1) | <0.001 |
| Chronic kidney disease stage V or End-stage renal failure (%) | 289 (19.5) | 1518 (1.0) | 1807 (1.2) | <0.001 |
| Depression (%) | 186 (12.5) | 2624 (1.8) | 2810 (1.9) | <0.001 |
| Schizophrenia (%) | 45 (3.0) | 516 (0.4) | 561 (0.4) | <0.001 |
| Dementia (%) | 62 (4.2) | 451 (0.3) | 513 (0.3) | <0.001 |
| Bipolar disease (%) | 4 (0.3) | 28 (0.02) | 32 (0.02) | <0.001 |
| Anxiety (%) | 63 (4.2) | 1227 (0.8) | 1290 (0.9) | <0.001 |
| Collagen vascular disease (%) | 54 (3.6) | 463 (0.3) | 517 (0.4) | <0.001 |
| Parkinson disease (%) | 38 (2.6) | 443 (0.3) | 481 (0.3) | <0.001 |
| Epilepsy (%) | 69 (4.6) | 646 (0.4) | 715 (0.5) | <0.001 |
| Coronary heart disease (%) | 463 (31.2) | 9046 (6.2) | 9509 (6.5) | <0.001 |
| Atrial fibrillation (%) | 127 (8.6) | 1159 (0.8) | 1286 (0.9) | <0.001 |
| Heart failure (%) | 279 (18.8) | 1917 (1.3) | 2196 (1.5) | <0.001 |
| Peripheral vascular disease (%) | 108 (7.3) | 1016 (0.7) | 1124 (0.8) | <0.001 |
| Hip fracture (%) | 28 (1.9) | 251 (0.2) | 279 (0.2) | <0.001 |
| Spine fracture (%) | 55 (3.7) | 397 (0.3) | 452 (0.3) | <0.001 |
| Chronic liver disease (%) | 94 (6.3) | 980 (0.7) | 1074 (0.7) | <0.001 |
| Pressure ulcer (%) | 55 (3.7) | 188 (0.1) | 243 (0.2) | <0.001 |
| Non-metastatic malignancy (%) | 172 (11.6) | 4712 (3.2) | 4884 (3.3) | <0.001 |
| Metastatic malignancy (%) | 59 (4.0) | 784 (0.5) | 843 (0.6) | <0.001 |
