# Supplementary material for: Outpatient primary and tertiary healthcare utilisation among public rental housing residents in Singapore
Source: BMC Health Serv Res. 2019 Apr 15;19:227. doi: 10.1186/s12913-019-4047-8 (PMC6466644; doi:10.1186/s12913-019-4047-8)
Supplement: Supplementary file 4 — Annex D. Patient characteristics and their association with frequent hospital admissions. Annex D shows the univariate analyses results for differences in characteristics of patients with frequent and non-frequent hospital admissions (DOCX 17 kb) [file 12913_2019_4047_MOESM4_ESM.docx]

Additional file 4

**Annex D:** Patient characteristics and their association with frequent hospital admissions

|  | **Frequent inpatient admitters**  **(n=1191)** | **Non-frequent inpatient admitters (n=145914)** | **All**  **(n=147105)** | **p value** |
| --- | --- | --- | --- | --- |
| **Patient Demographics** | | | | |
| Age, Mean (SD) | 60.86 (18.29) | 49.05 (17.19) | 50.2 (17.2) | <0.001 |
| Gender |  |  |  | 0.042 |
| Female (%) | 653 (54.8) | 84281 (57.8) | 84934 (57.7) |  |
| Male (%) | 538 (45.2) | 61633 (42.2) | 62171 (42.3) |  |
| Ethnicity |  |  |  | 0.003 |
| Chinese (%) | 887 (74.5) | 114569 (78.5) | 115456 (78.5) |  |
| Indian (%) | 113 (9.5) | 11150 (7.6) | 11263 (7.7) |  |
| Malay (%) | 145 (12.2) | 14437 (9.9) | 14582 (9.9) |  |
| Others (%) | 46 (3.9) | 5758 (3.9) | 5804 (3.9) |  |
| Resided in public rental housing | 218 (18.3) | 10182 (7.0) | 10400 (7.1) | <0.001 |
| **Medical Comorbidities** | | | | |
| Diabetes without complications (%) | 473 (39.7) | 20335 (13.9) | 20808 (14.1) | <0.001 |
| Hypertension (%) | 790 (66.3) | 42267 (29.0) | 43057 (29.3) | <0.001 |
| Hyperlipidemia (%) | 685 (57.5) | 41752 (28.6) | 42437 (28.8) | <0.001 |
| Asthma (%) | 130 (10.9) | 4828 (3.3) | 4958 (3.4) | <0.001 |
| Chronic Obstructive Pulmonary Disease (%) | 221 (18.6) | 2864 (2.0) | 3085 (2.1) | <0.001 |
| Chronic Obstructive Pulmonary Disease with cor pulmonale (%) | 218 (18.3) | 2356 (1.6) | 2574 (1.7) | <0.001 |
| Osteoarthritis (%) | 275 (23.1) | 16512 (11.3) | 16787 (11.4) | <0.001 |
| Hyperthyroidism (%) | 4 (0.3) | 1186 (0.8) | 1190 (0.8) | 0.072 |
| Hypothyroidism (%) | 30 (2.5) | 1884 (1.3) | 1914 (1.3) | 0.001 |
| Diabetes with complications (%) | 113 (9.5) | 2056 (1.4) | 2169 (1.5) | <0.001 |
| Cerebrovascular accident (%) | 260 (21.8) | 4913 (3.4) | 5173 (3.5) | <0.001 |
| Chronic Kidney Disease Stage 3-4 (%) | 352 (29.6) | 4262 (2.9) | 4614 (3.1) | <0.001 |
| Chronic kidney disease stage V or End-stage renal failure (%) | 335 (28.1) | 1472 (1.0) | 1807 (1.2) | <0.001 |
| Depression (%) | 143 (12.0) | 2667 (1.8) | 2810 (1.9) | <0.001 |
| Schizophrenia (%) | 26 (2.2) | 535 (0.4) | 561 (0.4) | <0.001 |
| Dementia (%) | 58 (4.9) | 455 (0.3) | 513 (0.3) | <0.001 |
| Bipolar disease (%) | 4 (0.3) | 28 (0.02) | 32 (0.02) | <0.001 |
| Anxiety (%) | 35 (2.9) | 1255 (0.9) | 1290 (0.9) | <0.001 |
| Collagen vascular disease (%) | 50 (4.2) | 467 (0.3) | 517 (0.4) | <0.001 |
| Parkinson disease (%) | 36 (3.0) | 445 (0.3) | 481 (0.3) | <0.001 |
| Epilepsy (%) | 52 (4.4) | 663 (0.5) | 715 (0.5) | <0.001 |
| Coronary heart disease (%) | 476 (40.0) | 9033 (6.2) | 9509 (6.5) | <0.001 |
| Atrial fibrillation (%) | 150 (12.6) | 1136 (0.8) | 1286 (0.9) | <0.001 |
| Heart failure (%) | 275 (23.1) | 1921 (1.3) | 2196 (1.5) | <0.001 |
| Peripheral vascular disease (%) | 140 (11.8) | 984 (0.7) | 1124 (0.8) | <0.001 |
| Hip fracture (%) | 30 (2.5) | 249 (0.2) | 279 (0.2) | <0.001 |
| Spine fracture (%) | 41 (3.4) | 411 (0.3) | 452 (0.3) | <0.001 |
| Chronic liver disease (%) | 99 (8.3) | 975 (0.7) | 1074 (0.7) | <0.001 |
| Pressure ulcer (%) | 59 (5.0) | 184 (0.1) | 243 (0.2) | <0.001 |
| Non-metastatic malignancy (%) | 286 (24.0) | 4598 (3.2) | 4884 (3.3) | <0.001 |
| Metastatic malignancy (%) | 116 (9.7) | 727 (0.5) | 843 (0.6) | <0.001 |
